# (±)-*trans*-5-Benzoyl-4-(3-bromo­phen­yl)-2-(1*H*-indol-3-yl)-4,5-dihydro­furan-3-carbonitrile

**DOI:** 10.1107/S1600536812030073

**Published:** 2012-07-10

**Authors:** J. Suresh, R. Vishnupriya, P. Gunasekaran, S. Perumal, P. L. Nilantha Lakshman

**Affiliations:** a Department of Physics, The Madura College, Madurai 625 011, India; b Department of Organic Chemistry, School of Chemistry, Madurai Kamaraj University, Madurai 625 021, India; c Department of Food Science and Technology, University of Ruhuna, Mapalana, Kamburupitiya 81100, Sri Lanka

## Abstract

The furan ring in the title compound, C_26_H_17_BrN_2_O_2_, adopts a twisted envelope conformation. The mol­ecular structure is stabilized by an intra­molecular C—H⋯O inter­action which generates an *S*(6) ring motif. The crystal packing is stabilized by N—H⋯O and C—H⋯Br inter­actions, generating an *R*_2_^2^(16) ring motif and a *C*(12) linear chain motif, respectively. Weak C—H⋯π bonding is also observed.

## Related literature

For the importance of furan derivatives, see: Auvin & Chabrier De Lassauniere (2005 ▶). For hydrogen-bonding motifs, see: Bernstein *et al.* (1995 ▶). For conformational analysis, see: Cremer & Pople (1975 ▶).
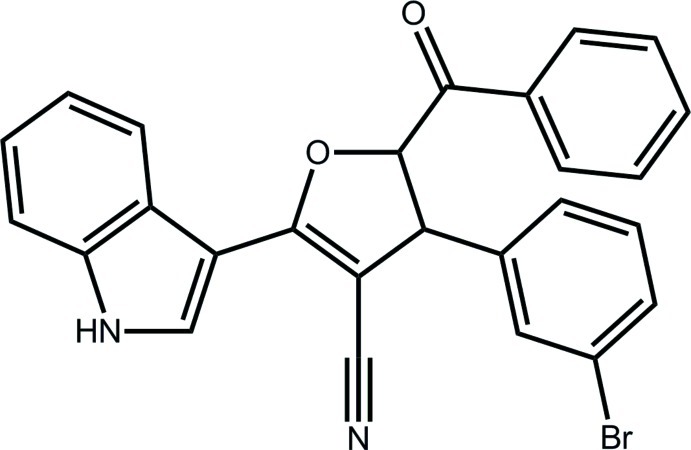


## Experimental

### 

#### Crystal data

C_26_H_17_BrN_2_O_2_
*M* *_r_* = 469.33
Monoclinic, 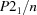

*a* = 9.8003 (6) Å
*b* = 15.8876 (10) Å
*c* = 13.5588 (9) Å
β = 100.306 (3)°
*V* = 2077.1 (2) Å^3^
*Z* = 4
Mo *K*α radiation
μ = 2.01 mm^−1^
*T* = 293 K
0.18 × 0.16 × 0.13 mm

#### Data collection

Bruker Kappa APEXII diffractometer
Absorption correction: multi-scan (*SADABS*; Sheldrick, 1996 ▶) *T*_min_ = 0.967, *T*_max_ = 0.974
17242 measured reflections
3635 independent reflections
2366 reflections with *I* > 2σ(*I*)
*R*_int_ = 0.044

#### Refinement

*R*[*F* ^2^ > 2σ(*F* ^2^)] = 0.043
*wR*(*F* ^2^) = 0.108
*S* = 1.02
3635 reflections
284 parameters
H atoms treated by a mixture of independent and constrained refinement
Δρ_max_ = 0.58 e Å^−3^
Δρ_min_ = −0.78 e Å^−3^

### 

Data collection: *APEX2* (Bruker, 2004 ▶); cell refinement: *SAINT* (Bruker, 2004 ▶); data reduction: *SAINT*; program(s) used to solve structure: *SHELXS97* (Sheldrick, 2008 ▶); program(s) used to refine structure: *SHELXL97* (Sheldrick, 2008 ▶); molecular graphics: *PLATON* (Spek, 2009 ▶); software used to prepare material for publication: *SHELXL97*.

## Supplementary Material

Crystal structure: contains datablock(s) global, I. DOI: 10.1107/S1600536812030073/tk5125sup1.cif

Structure factors: contains datablock(s) I. DOI: 10.1107/S1600536812030073/tk5125Isup2.hkl

Supplementary material file. DOI: 10.1107/S1600536812030073/tk5125Isup3.cml

Additional supplementary materials:  crystallographic information; 3D view; checkCIF report

## Figures and Tables

**Table 1:** Hydrogen-bond geometry (Å, °) *Cg*1 and *Cg*2 are the centroids of the C51–C56 and C32–C37 rings, respectively.

*D*—H⋯*A*	*D*—H	H⋯*A*	*D*⋯*A*	*D*—H⋯*A*
C33—H33⋯O1	0.93	2.48	2.995 (3)	115
N2—H2⋯O2^i^	0.81 (3)	2.26 (3)	3.006 (3)	153 (3)
C36—H36⋯Br1^ii^	0.93	2.87	3.509 (3)	127
C34—H34⋯*Cg*1^iii^	0.93	2.66	3.570 (3)	166
C47—H47⋯*Cg*2^iv^	0.93	2.95	3.784 (4)	149

Symmetry codes: (i) 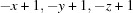; (ii) 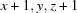; (iii) 
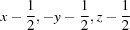
; (iv) 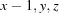.

**Fig. 1.**
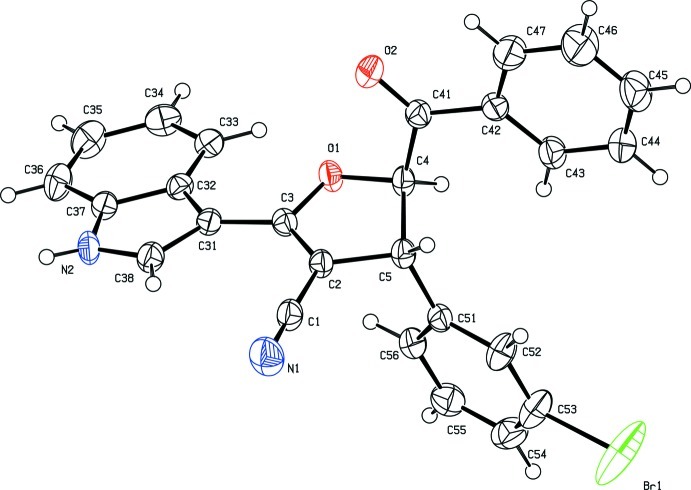
The molecular structure of (I), showing 40% probability displacement ellipsoids and the atom-numbering scheme.

## supplementary crystallographic information

### Comment

Furan derivatives have calplain-inhibiting activity and are used in the preparation of medicaments for the treatment of inflammatory and immunological diseases, cardiovascular and cerebro-vascular diseases, disorders of the central or peripheral nervous system, cachexia, osteoporosis, muscular dystrophy, proliferative diseases, cataracts, rejection reactions following organ transplants and auto-immune and viral diseases (Auvin & Chabrier De Lassauniere, 2005). In view of the high medicinal value of these compounds in conjunction with our research interest, prompted us to synthesize and report the X-ray studies of the title compound in this paper.

In the title compound (Fig 1), the five-membered furan ring in the structure adopts a twisted envelope conformation, as evident from the puckering parameters (Cremer & Pople, 1975): Q = 0.209 (3) Å and φ = 303.7 (8)°. The five (N2/C38/C31/C32/C37) and six-membered (C32—C37) rings in the indole group are planar, with a dihedral angle of 0.53 (1)° between them. The dihedral angle between the phenyl rings (C42—C47 and C51—C56) is 25.01 (1)°.

The molecular structure is stabilized by an intramolecular C–H···O interaction which generates an *S*(6) ring motif (Bernstein *et al.*, 1995). The presence of N–H···O hydrogen bonds leads to inversion dimers which are stabilized in the crystal packing by C–H···Br and C–H···π interactions, Table 1.

### Experimental

To a stirred mixture of 3-(3-bromophenyl)-2-(1*H*-indole-3-carbonyl) acrylonitrile (1.0 eq.) and phenacylpyridinium bromide (1.0 eq.) in water (10 ml) was added drop-wise triethylamine (0.25 eq.) at room temperature. The resulting clear solution, that slowly became turbid, was stirred at room temperature for 1.2 h. Then the separated free flowing solid was filtered and washed with methanol (3 ml) to afford compound as pale-yellow solids. The product thus obtained was recrystallized from EtOH-EtOAc mixture (1:1 ratio *v*/*v*. ml) to give pure compound, as pale-yellow crystals. Melting point: 468 K. Yield: 91%.

### Refinement

H atoms were placed at calculated positions and allowed to ride on their carrier atoms with C—H = 0.93–0.98 Å, and with *U*_iso_ = 1.2*U*_eq_(C). The N-bound H atom was located in a difference Fourier map and refined freely.

### Crystal data

**Table:**

C_26_H_17_BrN_2_O_2_	*F*(000) = 952
*M**_r_* = 469.33	*D*_x_ = 1.501 Mg m^−^^3^
Monoclinic, *P*2_1_/*n*	Mo *K*α radiation, λ = 0.71073 Å
Hall symbol: -P 2yn	Cell parameters from 2000 reflections
*a* = 9.8003 (6) Å	θ = 2.4–25°
*b* = 15.8876 (10) Å	µ = 2.01 mm^−^^1^
*c* = 13.5588 (9) Å	*T* = 293 K
β = 100.306 (3)°	Block, pale-yellow
*V* = 2077.1 (2) Å^3^	0.18 × 0.16 × 0.13 mm
*Z* = 4	

### Data collection

**Table:**

Bruker Kappa APEXII diffractometer	3635 independent reflections
Radiation source: fine-focus sealed tube	2366 reflections with *I* > 2σ(*I*)
Graphite monochromator	*R*_int_ = 0.044
Detector resolution: 0 pixels mm^-1^	θ_max_ = 25.0°, θ_min_ = 2.4°
ω and φ scans	*h* = −7→11
Absorption correction: multi-scan (*SADABS*; Sheldrick, 1996)	*k* = −18→18
*T*_min_ = 0.967, *T*_max_ = 0.974	*l* = −16→16
17242 measured reflections	

### Refinement

**Table:**

Refinement on *F*^2^	Primary atom site location: structure-invariant direct methods
Least-squares matrix: full	Secondary atom site location: difference Fourier map
*R*[*F*^2^ > 2σ(*F*^2^)] = 0.043	Hydrogen site location: inferred from neighbouring sites
*wR*(*F*^2^) = 0.108	H atoms treated by a mixture of independent and constrained refinement
*S* = 1.02	*w* = 1/[σ^2^(*F*_o_^2^) + (0.0397*P*)^2^ + 1.5055*P*] where *P* = (*F*_o_^2^ + 2*F*_c_^2^)/3
3635 reflections	(Δ/σ)_max_ < 0.001
284 parameters	Δρ_max_ = 0.58 e Å^−^^3^
0 restraints	Δρ_min_ = −0.78 e Å^−^^3^

### Special details

**Table:**

Geometry. All e.s.d.'s (except the e.s.d. in the dihedral angle between two l.s. planes) are estimated using the full covariance matrix. The cell e.s.d.'s are taken into account individually in the estimation of e.s.d.'s in distances, angles and torsion angles; correlations between e.s.d.'s in cell parameters are only used when they are defined by crystal symmetry. An approximate (isotropic) treatment of cell e.s.d.'s is used for estimating e.s.d.'s involving l.s. planes.
Refinement. Refinement of *F*^2^ against ALL reflections. The weighted *R*-factor *wR* and goodness of fit *S* are based on *F*^2^, conventional *R*-factors *R* are based on *F*, with *F* set to zero for negative *F*^2^. The threshold expression of *F*^2^ > σ(*F*^2^) is used only for calculating *R*-factors(gt) *etc*. and is not relevant to the choice of reflections for refinement. *R*-factors based on *F*^2^ are statistically about twice as large as those based on *F*, and *R*- factors based on ALL data will be even larger.

### Fractional atomic coordinates and isotropic or equivalent isotropic displacement parameters (Å^2^)

**Table:**

	*x*	*y*	*z*	*U*_iso_*/*U*_eq_	
H2	0.724 (3)	0.5931 (19)	0.496 (2)	0.038 (10)*	
Br1	0.09434 (6)	0.43918 (4)	−0.23671 (3)	0.1182 (3)	
O1	0.37349 (19)	0.36776 (12)	0.29701 (14)	0.0353 (5)	
O2	0.1364 (2)	0.35231 (14)	0.36843 (15)	0.0477 (6)	
N2	0.6871 (3)	0.55400 (18)	0.4627 (2)	0.0397 (7)	
C53	0.2243 (4)	0.4273 (2)	−0.1174 (2)	0.0546 (9)	
C41	0.1258 (3)	0.34022 (18)	0.2795 (2)	0.0342 (7)	
C32	0.6256 (3)	0.41997 (18)	0.4271 (2)	0.0311 (7)	
C42	−0.0082 (3)	0.31506 (18)	0.2155 (2)	0.0344 (7)	
C4	0.2472 (3)	0.35510 (18)	0.2255 (2)	0.0322 (7)	
H4	0.2578	0.3069	0.1825	0.039*	
C38	0.5653 (3)	0.55612 (19)	0.3984 (2)	0.0374 (7)	
H38	0.5176	0.6048	0.3751	0.045*	
C2	0.3141 (3)	0.49622 (17)	0.2325 (2)	0.0308 (7)	
C31	0.5221 (3)	0.47582 (18)	0.3725 (2)	0.0301 (7)	
C51	0.2728 (3)	0.42821 (17)	0.0617 (2)	0.0331 (7)	
C1	0.3123 (3)	0.5839 (2)	0.2189 (2)	0.0376 (7)	
C34	0.7591 (3)	0.3023 (2)	0.4948 (2)	0.0459 (8)	
H34	0.7713	0.2443	0.5003	0.055*	
C37	0.7273 (3)	0.47240 (19)	0.4816 (2)	0.0342 (7)	
C5	0.2256 (3)	0.43649 (17)	0.1618 (2)	0.0316 (7)	
H5	0.1281	0.4539	0.1518	0.038*	
C43	−0.0195 (3)	0.2963 (2)	0.1148 (2)	0.0418 (8)	
H43	0.0592	0.2981	0.0853	0.050*	
C3	0.4017 (3)	0.45201 (18)	0.3016 (2)	0.0313 (7)	
C52	0.1805 (3)	0.4376 (2)	−0.0265 (2)	0.0459 (8)	
H52	0.0884	0.4509	−0.0252	0.055*	
C44	−0.1450 (3)	0.2749 (2)	0.0575 (2)	0.0479 (9)	
H44	−0.1512	0.2633	−0.0104	0.057*	
C56	0.4098 (3)	0.4092 (2)	0.0573 (2)	0.0412 (8)	
H56	0.4742	0.4037	0.1162	0.049*	
C47	−0.1259 (3)	0.3112 (2)	0.2575 (3)	0.0585 (10)	
H47	−0.1209	0.3240	0.3250	0.070*	
C36	0.8450 (3)	0.4407 (2)	0.5426 (2)	0.0469 (9)	
H36	0.9115	0.4763	0.5783	0.056*	
C33	0.6428 (3)	0.33344 (19)	0.4342 (2)	0.0365 (7)	
H33	0.5771	0.2973	0.3988	0.044*	
C54	0.3588 (4)	0.4073 (2)	−0.1217 (3)	0.0538 (9)	
H54	0.3871	0.4000	−0.1830	0.065*	
C35	0.8592 (3)	0.3552 (2)	0.5481 (3)	0.0524 (9)	
H35	0.9371	0.3319	0.5881	0.063*	
N1	0.3062 (3)	0.65498 (19)	0.2061 (2)	0.0598 (8)	
C45	−0.2606 (4)	0.2708 (2)	0.1007 (3)	0.0601 (10)	
H45	−0.3457	0.2561	0.0625	0.072*	
C55	0.4510 (3)	0.3983 (2)	−0.0336 (3)	0.0508 (9)	
H55	0.5428	0.3846	−0.0354	0.061*	
C46	−0.2505 (4)	0.2885 (3)	0.2002 (3)	0.0742 (12)	
H46	−0.3291	0.2851	0.2297	0.089*	

### Atomic displacement parameters (Å^2^)

**Table:**

	*U*^11^	*U*^22^	*U*^33^	*U*^12^	*U*^13^	*U*^23^
Br1	0.1313 (5)	0.1734 (6)	0.0363 (3)	0.0828 (4)	−0.0219 (3)	−0.0174 (3)
O1	0.0322 (11)	0.0287 (12)	0.0400 (12)	−0.0040 (9)	−0.0073 (9)	0.0007 (9)
O2	0.0534 (14)	0.0585 (15)	0.0302 (12)	−0.0101 (11)	0.0049 (10)	−0.0076 (10)
N2	0.0401 (16)	0.0376 (18)	0.0367 (15)	−0.0097 (14)	−0.0060 (12)	−0.0074 (13)
C53	0.071 (3)	0.056 (2)	0.0317 (18)	0.0146 (19)	−0.0045 (17)	−0.0030 (16)
C41	0.0402 (18)	0.0276 (17)	0.0332 (18)	−0.0026 (13)	0.0019 (14)	−0.0010 (13)
C32	0.0306 (16)	0.0357 (18)	0.0275 (15)	0.0005 (14)	0.0062 (13)	0.0003 (13)
C42	0.0328 (16)	0.0336 (18)	0.0358 (17)	−0.0035 (14)	0.0032 (14)	0.0012 (13)
C4	0.0301 (16)	0.0313 (17)	0.0317 (16)	−0.0021 (13)	−0.0040 (13)	−0.0039 (13)
C38	0.0384 (18)	0.038 (2)	0.0338 (16)	−0.0001 (14)	−0.0002 (14)	−0.0003 (14)
C2	0.0329 (16)	0.0280 (17)	0.0298 (16)	−0.0018 (13)	0.0012 (13)	−0.0035 (13)
C31	0.0321 (16)	0.0297 (17)	0.0276 (15)	−0.0005 (13)	0.0028 (13)	−0.0014 (13)
C51	0.0383 (17)	0.0269 (16)	0.0323 (16)	−0.0053 (13)	0.0011 (13)	−0.0015 (12)
C1	0.0363 (18)	0.038 (2)	0.0350 (17)	−0.0017 (16)	−0.0027 (14)	−0.0018 (15)
C34	0.050 (2)	0.041 (2)	0.0476 (19)	0.0092 (17)	0.0098 (17)	0.0072 (16)
C37	0.0335 (17)	0.0386 (19)	0.0296 (16)	−0.0027 (14)	0.0035 (13)	−0.0019 (14)
C5	0.0290 (15)	0.0297 (17)	0.0335 (16)	0.0006 (13)	−0.0011 (13)	−0.0022 (13)
C43	0.0348 (18)	0.048 (2)	0.0419 (18)	−0.0074 (15)	0.0036 (15)	−0.0050 (15)
C3	0.0322 (16)	0.0288 (17)	0.0324 (16)	−0.0029 (13)	0.0049 (13)	−0.0036 (13)
C52	0.047 (2)	0.051 (2)	0.0349 (18)	0.0143 (16)	−0.0045 (15)	−0.0044 (15)
C44	0.046 (2)	0.053 (2)	0.0420 (19)	−0.0076 (17)	−0.0018 (16)	−0.0031 (16)
C56	0.0344 (18)	0.048 (2)	0.0392 (18)	−0.0086 (15)	0.0019 (14)	−0.0010 (15)
C47	0.045 (2)	0.088 (3)	0.044 (2)	−0.0136 (19)	0.0106 (17)	−0.0030 (19)
C36	0.0358 (18)	0.060 (2)	0.0404 (19)	0.0025 (16)	−0.0059 (15)	−0.0049 (17)
C33	0.0387 (18)	0.0374 (19)	0.0334 (16)	0.0008 (15)	0.0062 (14)	0.0008 (14)
C54	0.073 (3)	0.051 (2)	0.041 (2)	0.0002 (19)	0.0190 (19)	−0.0006 (16)
C35	0.040 (2)	0.065 (3)	0.048 (2)	0.0150 (18)	−0.0030 (16)	0.0059 (18)
N1	0.069 (2)	0.0371 (19)	0.067 (2)	−0.0010 (16)	−0.0045 (16)	0.0038 (16)
C45	0.037 (2)	0.070 (3)	0.067 (3)	−0.0065 (18)	−0.0064 (19)	−0.001 (2)
C55	0.043 (2)	0.058 (2)	0.053 (2)	−0.0068 (17)	0.0142 (18)	−0.0010 (18)
C46	0.038 (2)	0.120 (4)	0.066 (3)	−0.015 (2)	0.012 (2)	−0.006 (3)

### Geometric parameters (Å, º)

**Table:**

Br1—C53	1.881 (3)	C51—C5	1.516 (4)
O1—C3	1.366 (3)	C1—N1	1.143 (4)
O1—C4	1.443 (3)	C34—C33	1.372 (4)
O2—C41	1.207 (3)	C34—C35	1.392 (5)
N2—C38	1.347 (4)	C34—H34	0.9300
N2—C37	1.366 (4)	C37—C36	1.388 (4)
N2—H2	0.81 (3)	C5—H5	0.9800
C53—C54	1.367 (5)	C43—C44	1.375 (4)
C53—C52	1.386 (5)	C43—H43	0.9300
C41—C42	1.493 (4)	C52—H52	0.9300
C41—C4	1.523 (4)	C44—C45	1.367 (5)
C32—C33	1.386 (4)	C44—H44	0.9300
C32—C37	1.404 (4)	C56—C55	1.375 (4)
C32—C31	1.448 (4)	C56—H56	0.9300
C42—C47	1.376 (4)	C47—C46	1.374 (5)
C42—C43	1.383 (4)	C47—H47	0.9300
C4—C5	1.548 (4)	C36—C35	1.367 (5)
C4—H4	0.9800	C36—H36	0.9300
C38—C31	1.370 (4)	C33—H33	0.9300
C38—H38	0.9300	C54—C55	1.369 (5)
C2—C3	1.350 (4)	C54—H54	0.9300
C2—C1	1.405 (4)	C35—H35	0.9300
C2—C5	1.508 (4)	C45—C46	1.365 (5)
C31—C3	1.432 (4)	C45—H45	0.9300
C51—C52	1.373 (4)	C55—H55	0.9300
C51—C56	1.387 (4)	C46—H46	0.9300
			
C3—O1—C4	108.0 (2)	C51—C5—C4	113.2 (2)
C38—N2—C37	109.7 (3)	C2—C5—H5	110.3
C38—N2—H2	126 (2)	C51—C5—H5	110.3
C37—N2—H2	123 (2)	C4—C5—H5	110.3
C54—C53—C52	121.3 (3)	C44—C43—C42	121.1 (3)
C54—C53—Br1	119.8 (3)	C44—C43—H43	119.4
C52—C53—Br1	118.9 (3)	C42—C43—H43	119.4
O2—C41—C42	122.2 (3)	C2—C3—O1	112.3 (2)
O2—C41—C4	121.4 (3)	C2—C3—C31	132.4 (3)
C42—C41—C4	116.3 (2)	O1—C3—C31	115.2 (2)
C33—C32—C37	119.0 (3)	C51—C52—C53	120.1 (3)
C33—C32—C31	135.2 (3)	C51—C52—H52	120.0
C37—C32—C31	105.8 (2)	C53—C52—H52	120.0
C47—C42—C43	118.4 (3)	C45—C44—C43	119.7 (3)
C47—C42—C41	119.1 (3)	C45—C44—H44	120.1
C43—C42—C41	122.6 (3)	C43—C44—H44	120.1
O1—C4—C41	110.4 (2)	C55—C56—C51	120.6 (3)
O1—C4—C5	105.9 (2)	C55—C56—H56	119.7
C41—C4—C5	110.9 (2)	C51—C56—H56	119.7
O1—C4—H4	109.9	C46—C47—C42	120.3 (3)
C41—C4—H4	109.9	C46—C47—H47	119.9
C5—C4—H4	109.9	C42—C47—H47	119.9
N2—C38—C31	109.9 (3)	C35—C36—C37	117.3 (3)
N2—C38—H38	125.0	C35—C36—H36	121.3
C31—C38—H38	125.0	C37—C36—H36	121.3
C3—C2—C1	126.8 (3)	C34—C33—C32	118.5 (3)
C3—C2—C5	109.7 (2)	C34—C33—H33	120.7
C1—C2—C5	123.2 (3)	C32—C33—H33	120.7
C38—C31—C3	126.7 (3)	C53—C54—C55	118.6 (3)
C38—C31—C32	106.4 (2)	C53—C54—H54	120.7
C3—C31—C32	126.8 (3)	C55—C54—H54	120.7
C52—C51—C56	118.5 (3)	C36—C35—C34	121.1 (3)
C52—C51—C5	120.7 (3)	C36—C35—H35	119.4
C56—C51—C5	120.7 (3)	C34—C35—H35	119.4
N1—C1—C2	177.7 (3)	C46—C45—C44	119.7 (3)
C33—C34—C35	121.7 (3)	C46—C45—H45	120.1
C33—C34—H34	119.2	C44—C45—H45	120.1
C35—C34—H34	119.2	C54—C55—C56	120.9 (3)
N2—C37—C36	129.6 (3)	C54—C55—H55	119.5
N2—C37—C32	108.1 (3)	C56—C55—H55	119.5
C36—C37—C32	122.3 (3)	C45—C46—C47	120.8 (3)
C2—C5—C51	113.0 (2)	C45—C46—H46	119.6
C2—C5—C4	99.5 (2)	C47—C46—H46	119.6
			
O2—C41—C42—C47	4.7 (4)	C47—C42—C43—C44	0.5 (5)
C4—C41—C42—C47	−171.8 (3)	C41—C42—C43—C44	−178.7 (3)
O2—C41—C42—C43	−176.1 (3)	C1—C2—C3—O1	178.2 (3)
C4—C41—C42—C43	7.5 (4)	C5—C2—C3—O1	−8.3 (3)
C3—O1—C4—C41	−102.4 (2)	C1—C2—C3—C31	−6.9 (5)
C3—O1—C4—C5	17.7 (3)	C5—C2—C3—C31	166.6 (3)
O2—C41—C4—O1	11.2 (4)	C4—O1—C3—C2	−6.4 (3)
C42—C41—C4—O1	−172.3 (2)	C4—O1—C3—C31	177.8 (2)
O2—C41—C4—C5	−105.8 (3)	C38—C31—C3—C2	11.8 (5)
C42—C41—C4—C5	70.7 (3)	C32—C31—C3—C2	−164.9 (3)
C37—N2—C38—C31	−0.4 (3)	C38—C31—C3—O1	−173.4 (3)
N2—C38—C31—C3	−176.2 (3)	C32—C31—C3—O1	9.9 (4)
N2—C38—C31—C32	1.0 (3)	C56—C51—C52—C53	0.6 (5)
C33—C32—C31—C38	179.8 (3)	C5—C51—C52—C53	−178.0 (3)
C37—C32—C31—C38	−1.2 (3)	C54—C53—C52—C51	0.3 (5)
C33—C32—C31—C3	−3.0 (5)	Br1—C53—C52—C51	179.1 (2)
C37—C32—C31—C3	176.0 (3)	C42—C43—C44—C45	−1.0 (5)
C38—N2—C37—C36	179.8 (3)	C52—C51—C56—C55	−1.3 (5)
C38—N2—C37—C32	−0.4 (3)	C5—C51—C56—C55	177.3 (3)
C33—C32—C37—N2	−179.8 (3)	C43—C42—C47—C46	0.5 (5)
C31—C32—C37—N2	1.0 (3)	C41—C42—C47—C46	179.8 (3)
C33—C32—C37—C36	0.0 (4)	N2—C37—C36—C35	179.8 (3)
C31—C32—C37—C36	−179.2 (3)	C32—C37—C36—C35	0.0 (5)
C3—C2—C5—C51	−102.5 (3)	C35—C34—C33—C32	−0.4 (4)
C1—C2—C5—C51	71.3 (3)	C37—C32—C33—C34	0.2 (4)
C3—C2—C5—C4	17.8 (3)	C31—C32—C33—C34	179.1 (3)
C1—C2—C5—C4	−168.4 (3)	C52—C53—C54—C55	−0.6 (5)
C52—C51—C5—C2	−129.2 (3)	Br1—C53—C54—C55	−179.4 (3)
C56—C51—C5—C2	52.2 (4)	C37—C36—C35—C34	−0.2 (5)
C52—C51—C5—C4	118.7 (3)	C33—C34—C35—C36	0.4 (5)
C56—C51—C5—C4	−60.0 (3)	C43—C44—C45—C46	0.4 (6)
O1—C4—C5—C2	−20.8 (3)	C53—C54—C55—C56	−0.1 (5)
C41—C4—C5—C2	99.0 (2)	C51—C56—C55—C54	1.1 (5)
O1—C4—C5—C51	99.3 (3)	C44—C45—C46—C47	0.7 (6)
C41—C4—C5—C51	−140.9 (2)	C42—C47—C46—C45	−1.1 (6)

### Hydrogen-bond geometry (Å, º)

**Table:**

*D*—H···*A*	*D*—H	H···*A*	*D*···*A*	*D*—H···*A*
C33—H33···O1	0.93	2.48	2.995 (3)	115
N2—H2···O2^i^	0.81 (3)	2.26 (3)	3.006 (3)	153 (3)
C36—H36···Br1^ii^	0.93	2.87	3.509 (3)	127
C34—H34···*Cg*1^iii^	0.93	2.66	3.570 (3)	166
C47—H47···*Cg*2^iv^	0.93	2.95	3.784 (4)	149

Symmetry codes: (i) −*x*+1, −*y*+1, −*z*+1; (ii) *x*+1, *y*, *z*+1; (iii) *x*−1/2, −*y*−1/2, *z*−1/2; (iv) *x*−1, *y*, *z*.
